# Identification on mitogen-activated protein kinase signaling cascades by integrating protein interaction with transcriptional profiling analysis in cotton

**DOI:** 10.1038/s41598-018-26400-w

**Published:** 2018-05-25

**Authors:** Xueying Zhang, Xinyue Mi, Chuan Chen, Haitang Wang, Wangzhen Guo

**Affiliations:** State Key Laboratory of Crop Genetics & Germplasm Enhancement, Hybrid Cotton R & D Engineering Research Center, Ministry of Education, Nanjing Agricultural University, Nanjing, 210095 China

## Abstract

Plant mitogen-activated protein kinase (MAPK) cascades play important roles in development and stress responses. In previous studies, we have systematically investigated the mitogen-activated protein kinase kinase (MKK) and MAPK gene families in cotton. However, the complete interactions between MAPK gene family members in MAPK signaling cascade is poorly characterized. Herein, we investigated the mitogen-activated protein kinase kinase kinase (MAPKKK) family members and identified a total of 89 MAPKKK genes in the *Gossypium raimondii* genome. We cloned 51 MAPKKKs in *G*. *hirsutum* and investigated the interactions between MKK and MAPKKK proteins through yeast-two hybrid assays. A total of 18 interactive protein pairs involved in 14 MAPKKKs and six MKKs were found. Among these, 13 interactive pairs had not been reported previously. Gene expression patterns revealed that 12 MAPKKKs were involved in diverse signaling pathways triggered by hormone treatments or abiotic stresses. By combining the MKK-MAPK and MKK-MAPKKK protein interactions with gene expression patterns, 38 potential MAPK signaling modules involved in the complicated cross-talks were identified, which provide a basis on elucidating biological function of the MAPK cascade in response to hormonal and/or stress responses. The systematic investigation in MAPK signaling cascades will lay a foundation for understanding the functional roles of different MAPK cascades in signal transduction pathways, and for the improvement of various defense responses in cotton.

## Introduction

Mitogen-activated protein kinase (MAPK) cascades play central roles in the signaling pathways that transduce extracellular stimuli into intracellular responses in eukaryotes^1–3^. The classical MAPK cascade consists of three distinct modules: mitogen-activated protein kinases (MAPKs), mitogen-activated protein kinase kinases (MAPKKs/MKKs), and mitogen-activated protein kinase kinase kinases (MAPKKKs/MEKKs)^4^. As the first components of MAPK cascades, MAPKKKs phosphorylate and activate MKKs, MKKs then phosphorylate MAPKs, and MAPKs can phosphorylate a wide range of substrates, such as transcription factors, kinases and cytoskeleton associated proteins. These signaling cascades have frequent crosstalk to allow plants to regulate both development and stress tolerance^5^.

As the first member of the MAPK signaling cascade, MAPKKKs have been identified in various plant genomes. Compared to MAPK and MAPKK, MAPKKK is a large gene family which includes 80 putative genes in *Arabidopsis*^2,6^, 75 in rice (*Oryza sativa*)^7–9^, 74 in maize (*Zea mays*)^10^, 59 in cucumber (*Cucumis sativus*)^11^, 75 in *Brachypodium distachyon*, and 150 in soybean (*Glycine max*)^12,13^. Based on the catalytic kinase domains, MAPKKKs can be classified into three groups: MEKK-like subfamily, Raf-like subfamily and ZIK subfamily. In detail, the conserved catalytic domain and signature is G (T/S) PX (F/Y/W) MAPEV in the MEKK subfamily members, GTXX (W/Y) MAPE in the Raf-like MAPKKK members, and GTPEFMAPE (L/V/M) (Y/F/L) in the ZIK subfamily^2,14,15^. In addition, Raf and ZIK subfamily proteins contain a C-terminal kinase domain (KD) and a long N-terminal regulatory domain (RD) that might function as a scaffold to activate MAPKKs or other proteins in signaling cascades^16^. To date, several MAPKKKs have been characterized in *Arabidopsis*. For example, MEKK1 is involved in disease stress responses and contributes to the acquisition of freezing tolerance^17–19^; ANP2/3 play an important role in regulating plant cell division^20^; YODA participates in the regulation of stomatal development and dynamics^21^; Two members in the *Arabidopsis* Raf subfamily, *AtCTR1* and *AtEDR1* negatively regulate ethylene signaling transduction and are involved in pathogen resistance^22,23^; *Raf5* responds to salt stress, and silencing *Raf5* in *Arabidopsis* enhanced salt tolerance^24^. In addition, the functions of MAPKKK genes have also been studied in other species. For example, in rice, *MAPKKK6* contributes to the acquisition of dehydration tolerance through reactive oxygen species (ROS) scavenging^25^, and *ILA1* (*MAPKKK43*) plays important role in mechanical tissue formation at the leaf lamina joint^26^. In tomato, *SlMAPKKKα* and *SlMAPKKKε* positively regulate cell death and enhance plant immunity and disease resistance^27,28^. Only two Raf-like MAPKKK genes have been reported in cotton. *GhRaf19* is involved in positively regulating the tolerance to cold stress and negatively regulating to drought and salt by modulating ROS in cotton^29^. Overexpression of *GhMAPKKK40* in *nicotiana benthaminana* inhibited the transgenic plants growth and development and reduced the tolerance to biotic and abiotic stress^30^.

To date, few complete MAPK signaling cascades have been characterized in *Arabidopsis*. MEKK1-MKK1/2-MPK4 cascade is involved in responses to cold, salt and pathogen infection^18,19^; MAPKKK17/18-MKK3-MPK1/2/7/14 cascade is an abscisic acid (ABA) dependent MAPK pathway and plays a role in ABA stress signaling^31^; YDA-MKK4/5-MPK3/6 module is involved in the regulation of stomatal development^21^; and the ANP3-MKK6-MPK4 cascade participates in male-specific meiotic cytokinesis^20^. In previous studies, a total of 11 MKK genes and 28 MAPK genes were identified in *G*. *raimondii*^32,33^, however, no complete MAPK cascade has yet been reported in cotton. Here, we systematically investigated the MAPKKKs in *G*. *raimondii* and explored complete cascades in MAPK signaling networks via protein interaction and transcriptional profiling analysis in cotton. As a result, we identified 89 MAPKKK genes in *G*. *raimondii* and classified them according to their homology with MAPKKK genes in *Arabidopsis*. We analyzed their gene structures, chromosomal locations, duplicated genes and evolutionary mechanisms. We also cloned 51 MAPKKK genes from *G*. *hirsutum* and examined their interactions with downstream MKK proteins. By integrating the protein interaction data with transcriptional profiling analysis, we identified 38 MAPK signaling modules that are potentially involved in the complicated cross-talks of signal and abiotic stress responses. This work lays a solid foundation to better understand the molecular mechanisms regulated by MAPK cascades in cotton, and to be utilized for the improvement of cotton responses to stress in breeding.

## Results

### Identification of MAPKKK genes in cotton

The genome sequences of four sequenced cotton species (*G*. *raimondii*, *G*. *arboreum*, *G*. *hirsutum* acc. TM-1 and *G*. *barbadense* acc. 3–79) offer the information to identify MAPKKK gene members in this important crop^34–37^. In total, we characterized 89, 86, 173 and 173 MAPKKKs in *G*. *raimondii*, *G*. *arboreum*, *G*. *hirsutum* acc. TM-1 and *G*. *barbadense* acc. 3–79, respectively (Supplementary Dataset 1). From a phylogenetic viewpoint, one member in *G*. *raimondii*, should correspond to one homolog in *G*. *arboreum* and two homeologs, one from the A and one from the D subgenome, in the tetraploid species, *G*. *hirsutum* acc. TM-1 and *G*. *barbadense* acc. 3–79. Here, 75 members showed such a one-to-one correspondence. Inconsistencies in the other members might be due to the differences in sequencing methods, assembly inaccuracy in partial chromosomal regions, or structural variations during the evolution of *Gossypium*, and need to be further confirmed.

**Figure 1:**
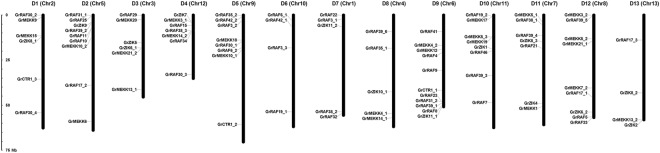
Chromosomal distribution of MAPKKK genes in *G*. *raimondii*. The 89 candidate MAPKKK genes were matched to 13 chromosome of the *G*. *raimondii* genome. The nomenclature of MAPKKK genes in *G*. *raimondii* follows its homology with *Arabidopsis* MAPKKK proteins. The chromosome numbers from D1 to D13, were consistent with the newly-updated interspecific genetic map of allotetraploid cultivated cotton species^38^. The names of the scaffolds from the *G*. *raimondii* genome are indicated in brackets.

The number of amino acids in the *G*. *raimondii* MAPKKKs ranged from 296 to 1428, with putative molecular weights (MWs) ranging from 33.82 to 156.99 KDa and isoelectric points (pIs) of 4.65 to 9.29. Detailed information, including the nomenclature, origins, chromosome locations, number of amino acids, pIs, MWs and subcellular localizations of these proteins, is listed in Supplementary Dataset 2. Chromosomal location anchored 89 MAPKKK genes to the 13 scaffolds of the *G*. *raimondii* genome^34^, renamed D1 to D13, by integrating the 13 scaffolds with the high-density interspecific genetic map of allotetraploid cultivated cotton species reported previously^38^. The number of MAPKKK genes in each chromosome differed, and ranged from four (D13) to 11 (D9) (Fig. 1).

**Figure 2:**
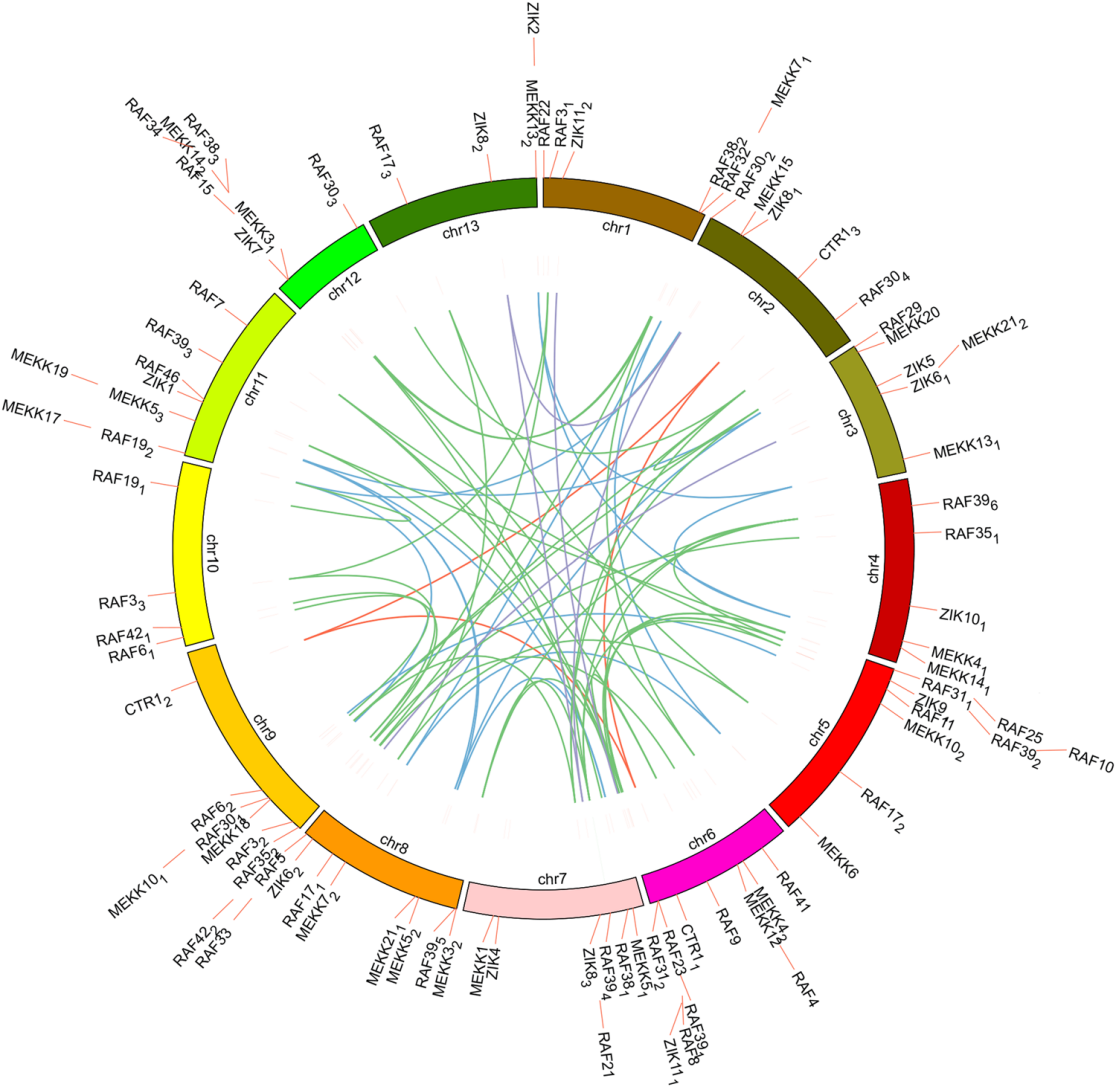
The intra-genomic comparison showed gene synteny of MAPKKK genes in *G*. *raimondii* (Gr). The light blue and green lines indicated the intra-genomic synteny of MEKK and Raf genes, and the purple lines for ZIK genes in *G*. *raimondii*. Chr1 to Chr13 was following the names of the scaffolds from the *G*. *raimondii* genome.

The *G*. *raimondii* genome underwent at least two rounds of genome-wide duplication^34^. To understand the mechanisms of expansion of the MAPKKK gene family in *G*. *raimondii*, tandem and segmental duplication events of MAPKKK gene family were investigated through genome synteny analysis. Among the 89 MAPKKKs in *G*. *raimondii*, we identified 75 genes within 65 pairs of synthenic blocks (Fig. 2; Supplementary Dataset 3), comprising 15 pairs in the MEKK-subfamily, 45 pairs in the Raf-subfamily and 5 pairs in the ZIK-subfamily. The ratios of non-synonymous substitution rate (Ka) to synonymous substitution rate (Ks) in the 65 pairs of paralog genes were less than 1, ranging from 0.0456 to 0.4588. This implies that these paralog gene pairs originating from *Gossypium* mainly experienced purifying selection after the segmental duplication, and their functions did not diverge much from each other during subsequent evolutionary processes.

### Phylogenetic and structural analyses of MAPKKKs in *G*. *raimondii*

**Figure 3:**
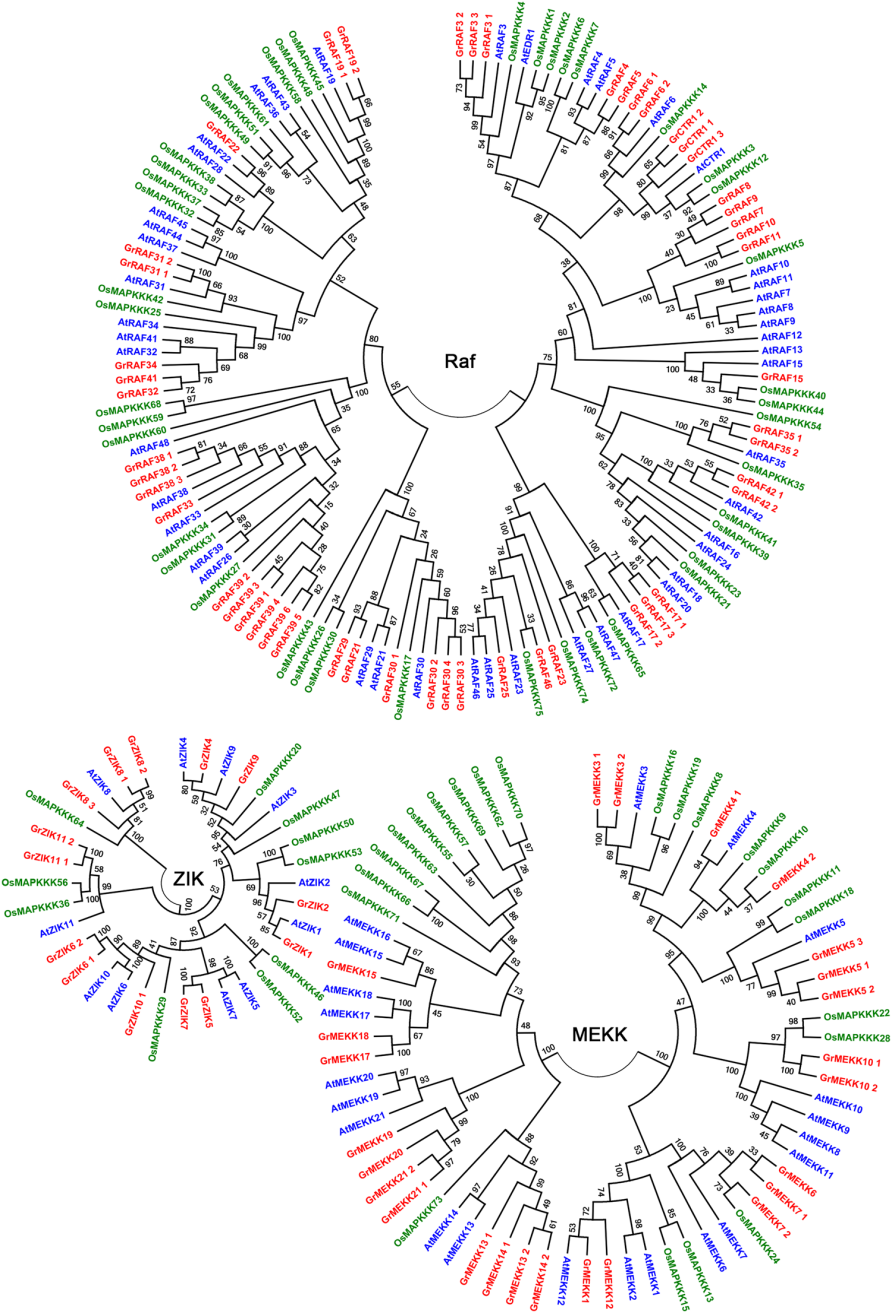
Phylogenetic tree of MAPKKK family genes from *G*. *raimondii*, *A*. *thaliana* and *O*. *sativa*. Amino acid sequences were aligned using Clustal W in MEGA 6 software and Maximum likelihood method was used to perform phylogenetic analysis with 1,000 bootstrap replicates. MAPKKKs from *G*. *raimondii*, *A*. *thaliana* and *O*. *sativa* were shown in red, blue, and green, respectively.

To evaluate the phylogenetic relationships between the MAPKKKs in *G*. *raimondii* and those in other species, 80 MAPKKKs in *Arabidopsis*, 75 in *O*. *sativa* and 89 in *G*. *raimondii* were selected to construct the phylogenetic tree using the maximum likelihood method via MEGA6 (Fig. 3). All of the MAPKKKs could be clustered into three major groups, the MEKK, Raf and ZIK subfamilies. There were 21 MAPKKKs from *Arabidopsis*, 22 from *O*. *sativa* and 25 from *G*. *raimondii* grouped into the MEKK subfamily; 48 from *Arabidopsis*, 43 from *O*. *sativa* and 50 from *G*. *raimondii* in the Raf subfamily; and only 11 from *Arabidopsis*, 10 from *O*. *sativa* and 14 from *G*. *raimondii* in the ZIK family.

Alignment of amino acid sequences of MAPKKKs in *G*. *raimondii* revealed that those in the MEKK subfamily contained the G(T/S)PX(W/Y/F)MAPEV signature, those in the Raf subfamily contained GTXX(W/Y)MAPE, and those in the ZIK subfamily contained GTPEFMAPE(L/V)Y (Supplementary Fig. 1). Analysis of the exon positions and intron phases of the MAPKKK genes showed that those in the MEKK and Raf subfamilies had a more sophisticated structure than those in the ZIK subfamily. Introns in ZIK genes were ranging from 1 to 8. In the MEKK subfamily, 8 genes had only one intron, and others had 2 to 24 introns. In the Raf subfamily, 13 out 50 genes had six introns, and other Raf genes had 2 to 16 introns. Most of the MAPKKK genes were clustered together in the phylogenetic tree and shared similar exon/intron structures and intron phases, suggesting evolutionary conservation of the gene structure **(**Supplementary Fig. 2).

PlantsP databases were used to predict the protein domains of these MAPKKK genes in *G*. *raimondii*. Conserved domain analysis showed that all GrMAPKKK protein sequences contain a protein kinase domain. Most of them contained a serine/threonine protein kinase active site and an ATP binding site. In addition, the MAPKKKs also had some other conserved domains, such as the NLS-BP Bipartite nuclear localization signal and a C-terminal conserved region (Supplementary Fig. 2). The conserved domains may facilitate the identification of functional units in these kinases and will accelerate our understanding of their biological functions^39,40^.

### Spatial and temporal expression of MAPKKK genes in *G*. *hirsutum* acc. TM-1

**Figure 4:**
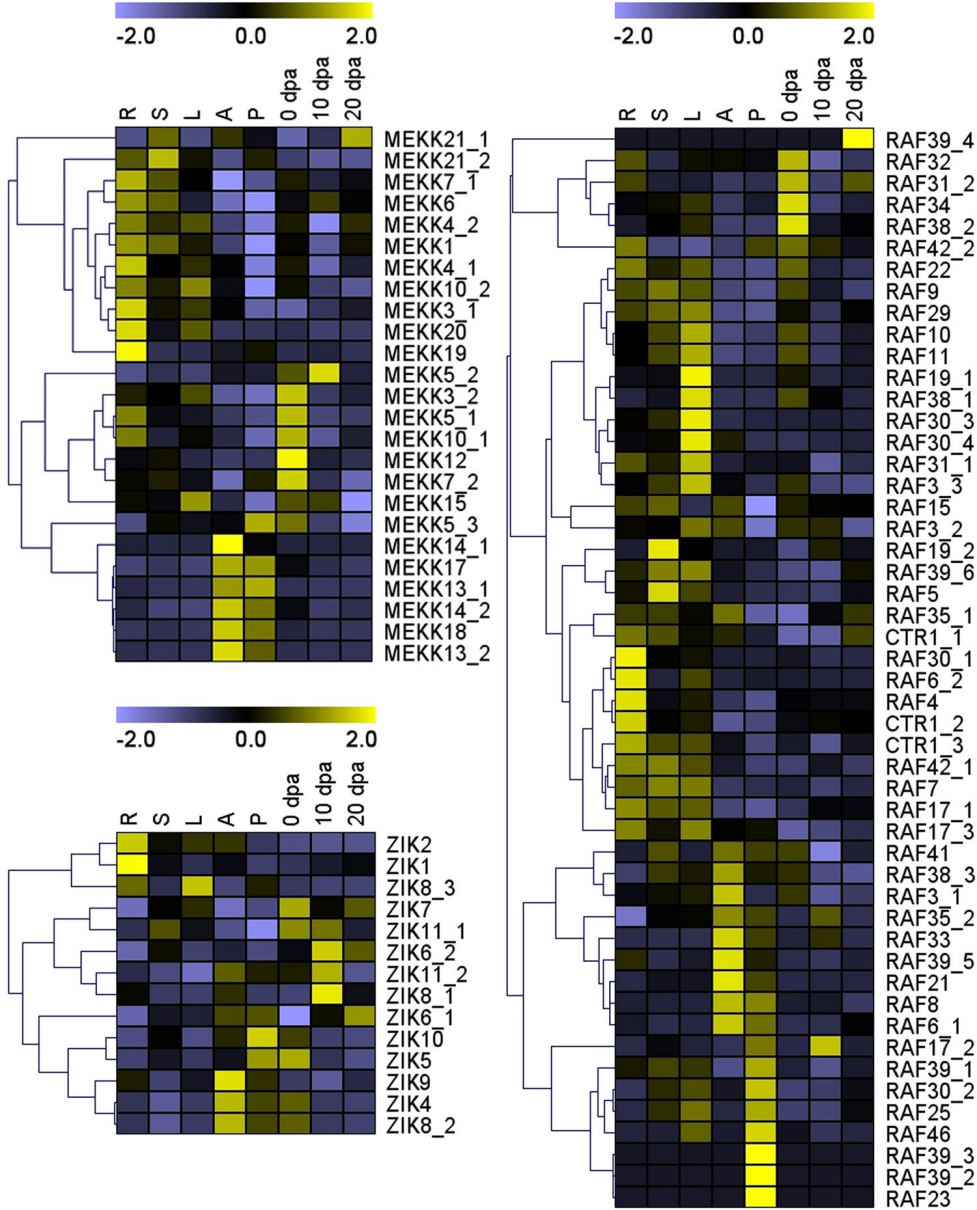
Expression pattern of 89 MAPKKK genes in *G*. *hirsutum* acc TM-1 based on transcriptome sequencing data. Expression pattern of the MAPKKKs in eight cotton tissues (root; stem; leaf; petal; anther; ovule at 0 dpa; fiber at 10 dpa and 20 dpa) were obtained with FPKM values and calculated by Z-Score. The RNA-Seq data used here could be downloaded from http://www.ncbi.nlm.nih.gov/bioproject/PRJNA248163/.

To elucidate the expression patterns of MAPKKK genes in cotton growth and development, we analyzed the expression of MAPKKKs in different tissues and organs of *G*. *hirsutum* acc. TM-1. As shown in Fig. 4, all MAPKKK members were expressed in *G*. *hirsutum* TM-1 organs at least one developmental stage. Analysis showed that 41 MAPKKK members, comprising 11 in the MEKK subfamily, 27 in the Raf subfamily and 3 in the ZIK subfamily, were more highly expressed in vegetative organs than in other organs. In addition, 29 MAPKKK members, comprising 7 in the MEKK subfamily, 17 in the Raf subfamily and 5 in the ZIK subfamily, had higher expression levels in petals or anthers than in other organs. A further 19 MAPKKK members, comprising 7 in the MEKK subfamily, 6 in the Raf subfamily and 6 in the ZIK subfamily, had higher expression levels in ovules and fibers than in other organs. Moreover, several paralogs (MEKK6 and MEKK7_1; MEKK13_1, MEKK13_2 and MEKK14_1; MEKK19 and MEKK20; CTR1_1, CTR1_2 and CTR1_3; RAF17_1 and RAF17_3; RAF23, RAF25 and RAF46; RAF30_3 and RAF30_4; RAF32 and RAF34; and ZIK11_1 and ZIK11_2) were found with the similar expression profiles, indicating their sub-functionalization during the evolutionary process. However, other gene pairs showed quite different expression patterns in the different developmental stages, implying that these genes had undergone functional differentiation.

### Analyses of the interactions between MAPKKK and MKK family members

**Figure 5:**
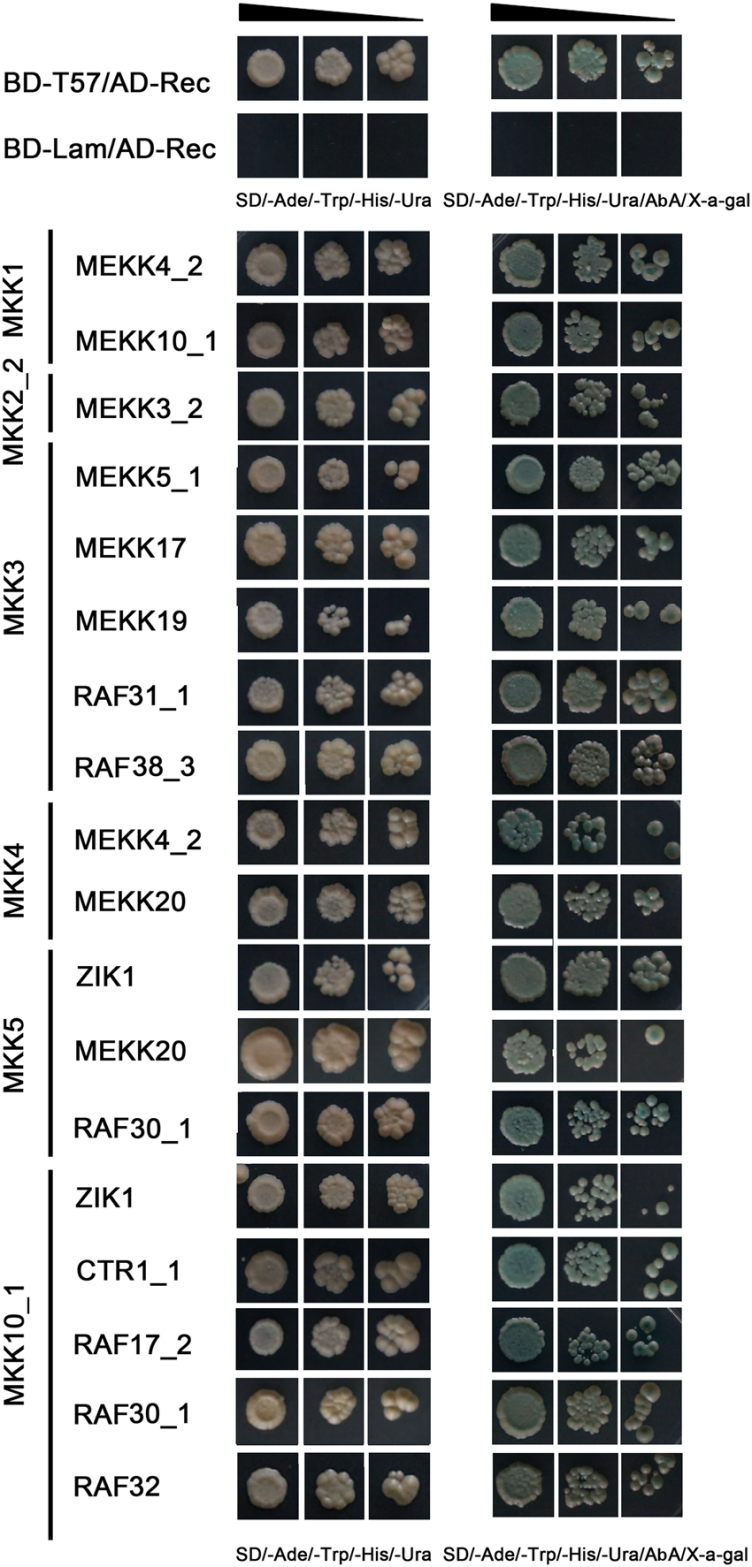
Comparative yeast two-hybrid (Y2H) interaction analyses of MAPKKKs with MKKs. Protein interactions between MAPKKKs and MKKs were shown. Yeasts harboring the indicated plasmid combinations were grown on selective medium SD/-Ade/-His/-Leu/-Trp, positive interactions was examined by addition of Aureobasidin A and X-α-gal. Positive (pGBKT7-53 + pGADT7-rec) and negative (pGBKT7-Lam + pGADT7-rec) as controls. Three independent co-transformation experiments showed the similar results.

To examine the regulatory network of MAPK cascade kinases, it is necessary to characterize the interactions between MAPKKKs and MKKs, as well as the interactions between MKKs and MAPKs in cotton. We have previously studied MKK/MAPK interaction partners in cotton^32^. Further analysis of the physical interactions between MAPKKKs and MKKs will help to clarify the MAPK signaling cascades. Therefore, we used a yeast two-hybrid (Y2H) system to detect interactions between MAPKKKs and MKKs. Due to the high similarity (more than 96%) in the amino acid sequences of MAPKKK homoeologs in the A- and D-subgenomes (Supplementary Dataset 4), we performed PCR-based cloning for either of the MAPKKK homoeologs. A total of 51 MAPKKKs were cloned and sequenced to confirm their complete open reading frames, comprising 12 in the MEKK-subfamily, 31 in the Raf-subfamily and 8 in the ZIK-subfamily (GenBank accession numbers: MF150710-MF150760). Eight MKKs had been cloned into pGBDT7 vectors in our previous study^32^, and a further 51 MAPKKKs here were cloned into pGADT7 vectors for MAPKKKs/MKKs Y2H analysis. A total of 18 interactions were identified by the Y2H assay (Fig. 5), which comprised 9 MEKK-MKK, 7 Raf-MKK and 2 ZIK-MKK interaction pairs. We also found that 6 MKKs interacted with at least one MAPKKK protein, while MKK6 and MKK7 did not interact with any of the MAPKKK proteins assayed. In addition, several MAPKKKs were not detected to interact with any of the MKK proteins. Their functional characteristics need to be further analyzed. Of the MAPKKK-MKK interaction pairs, MKK1 interacted with MEKK4_2 and MEKK10_1; while its sister, MKK2_2, did not show any affinity toward these proteins, but did interact with MEKK3_2, which indicates that the function of paralogous genes diverged during the evolutionary process. MKK3 interacted with 5 MAPKKKs, with 3 MEKKs (MEKK5_1, MEKK17 and MEKK19) and 2 Rafs (RAF31_1 and RAF38_3); while MKK4 and MKK5 both interacted with MEKK20, as well as other MAPKKKs. In addition, MKK4 interacted with MEKK4_2, MKK5 interacted with ZIK1 and RAF30_1, and MKK10_1 interacted ZIK1 and 4 Raf members (CTR1_1, RAF17_2, RAF30_1 and RAF32).

**Figure 6:**
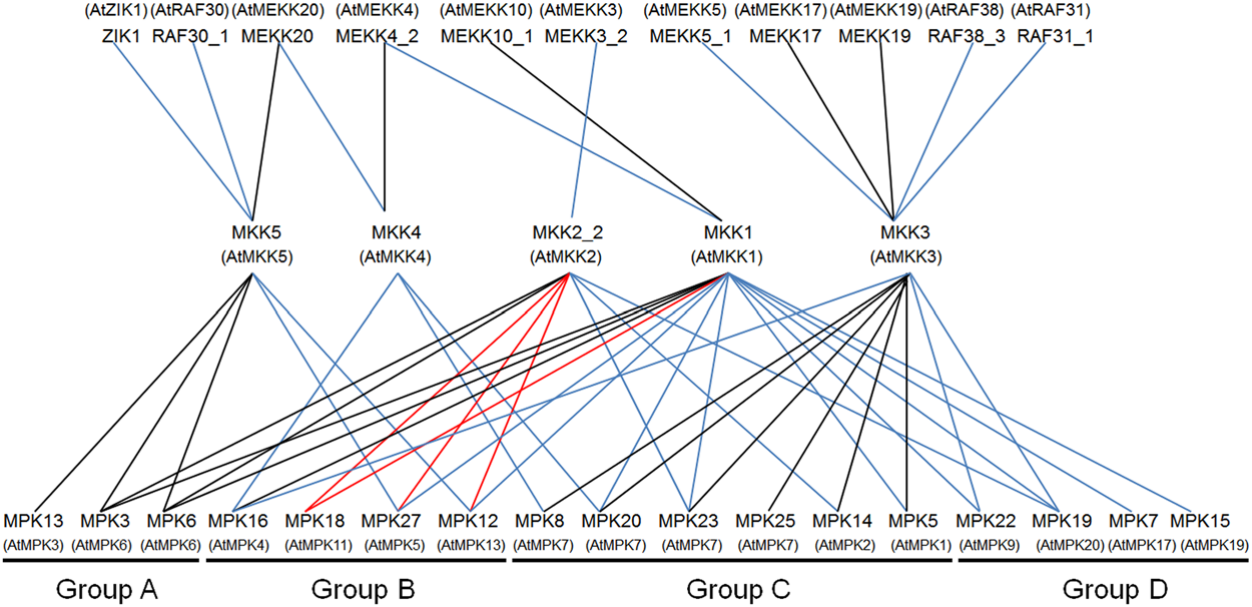
MAPK cascades in cotton compared to those in *Arabidopsis*. All the lines indicated the confirmed interactions of MAPKKK-MKK and MKK-MAPK in cotton. Black lines indicated the interactions reported with confirmed function under stress in *Arabidopsis*, which was also identified in cotton. Red lines indicated the shared interactions in *Arabidopsis* and cotton, but no functions were reported in *Arabidopsis*. Blue lines indicated that the interactions only confirmed in cotton without reported in *Arabidopsis*.

Among the 18 identified interaction pairs, five have been reported previously in *Arabidopsis*, and 13 interactions are novel combinations found in cotton. We found that individual MAPKKK proteins were able to interact with more than one MKK protein, and multiple MAPKKKs individually interacted with the same MKK. For example, MEKK20 interacted with both MKK4 and MKK5, which belong to the same subgroup, however, MEKK4_2 interacted with MKK1 and MKK4, which belong to different subgroups. Similarly, multiple MAPKKKs interacted with one MKK, also the MAPKKKs belonging to the same subgroup interacted with MKK1 (MEKK4_2-MKK1, MEKK10_1-MKK1) or MAPKKKs in the different subgroups interacted with MKK5 (ZIK1-MKK5, MEKK20-MKK5, RAF30_1-MKK5). The diverse interactions between MAPKKKs and MKKs highlight the functional diversity of members of the MAPK signal pathways (Fig. 6).

### Expression patterns of MAPKKK genes following multiple signal treatments and abiotic stresses

**Figure 7:**
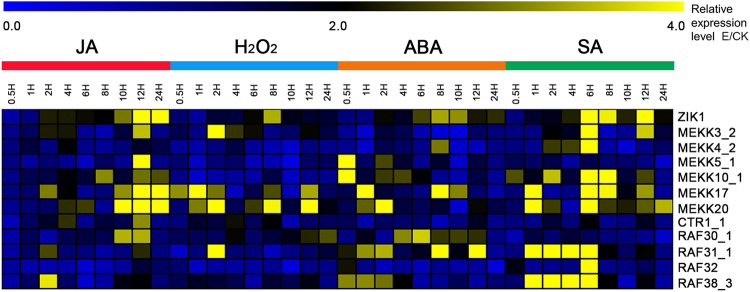
Expression pattern of MAPKKK genes under different stress-related signal treatments. The data are presented in clusters using the fold-change (E/C) of expression for the 12 investigated MAPKKK genes in response to stress-related signal treatments (Experiments (E): Inducers involved in JA, H_2_O_2_, ABA and SA), in comparison to their respective controls (C). Yellow color indicated that the MAPKKK genes were induced with more than 2-fold change in each experiment.

The last components of the MAPK cascade (MKK and MAPK) triggered in response to abiotic and biotic stresses have been characterized in our previous studies^32,33^. In the study, a total of 14 MAPKKKs were found to interact with MKKs, comprising seven MEKK members, six Raf members and one ZIK member. We conducted qRT-PCR analyses of each of these MAPKKK genes to examine their expression in response to four signal stresses, jasmonic acid (JA), hydrogen peroxide (H_2_O_2_), abscisic acid (ABA) and salicylic acid (SA) (Fig. 7, Supplementary Fig. 3), With the exception of MEKK19 and RAF17_2, having the low expression levels in leaf tissue and expression changes less than two-fold under the stresses compared to controls, other MAPKKK genes showed to be significantly induced in different stressors. Of them, ten MAPKKK genes were significantly up-regulated after JA treatment. Seven genes were induced and reached their highest levels at 12 h after treatment, including four MEKKs (MEKK3_2, MEKK5_1, MEKK17 and MEKK20) and three Rafs (CTR1_1, RAF30_1 and RAF32). ZIK1 was induced and its expression reached the highest level 24 h after treatment; and the expression of MEKK10_1 and RAF38_3 was induced and reached peaks 8 h and 2 h after treatment, respectively. After H_2_O_2_ treatment, seven MAPKKK genes, comprising three MEKKs (MEKK3_2, MEKK17 and MEKK20), three RAFs (CTR1_1, RAF30_1and RAF32) and ZIK1, were significantly up-regulated and their expression reached peaks at different time points. In addition, ten MAPKKK genes were induced by ABA treatment. Of them, two MEKKs (MEKK5_1 and MEKK10_1) and two Rafs (RAF32 and RAF38_3) were up-regulated and reached peaks quickly at 0.5 h. ZIK1, MEKK4_2 and MEKK17 were induced and their expression peaked at 8 h; MEKK20 and RAF32 were induced reached peak expression levels at 1 or 2 h; and RAF30_1 was induced and its expression reached a peak at 6 h. Finally, nine MAPKKK genes were significantly up-regulated and their expression reached peaks 6 h after SA treatment.

**Figure 8:**
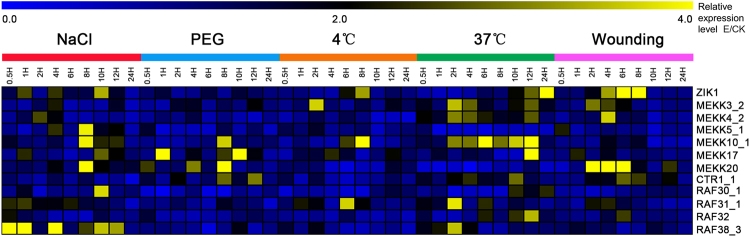
Expression pattern of MAPKKK genes under different stress treatments. The data are presented in clusters using the fold-change (E/C) of expression for the 12 investigated MAPKKK genes in response to stress treatments (Experiments (E): Inducers involved in NaCl, PEG, 4 °C, 37 °C and wounding), in comparison to their respective controls (C). Yellow color indicated that the MAPKKK genes were induced with more than 2-fold change in each experiment.

The expression of these 12 MAPKKK genes was investigated in response to several abiotic stresses: salinity, drought, cold, heat and wounding (Fig. 8, Supplementary Fig. 4). A total of 11 MAPKKKs were found to be up-regulated after NaCl treatment. Of them, three MAPKKKs (ZIK1, MEKK17 and RAF30_1) were induced and reached peak expression levels at 10 h, the expression of three MEKKs (MEKK5_1, MEKK10_1 and MEKK20) peaked at 8 h, the others (MEKK3_2, MEKK4_2, RAF31_1, RAF32 and RAF38_3) were significantly up-regulated and reached peak expression levels at different time points after NaCl treatment. Four MAPKKK genes were up-regulated by drought treatment, including MEKK17 and three MAPKKKs (MEKK10_1, MEKK20 and CTR1_1), and reached peak expression levels at 1 h and 8 h, respectively. Following temperature stress, five MAPKKK genes (ZIK1, MEKK3_2, MEKK10_1, MEKK17 and RAF32) were induced and were highly expressed upon exposure to low temperature treatment (4 °C) with diverse expression patterns. In addition, four (MEKK3_2, MEKK4_2, RAF32 and RAF38_3) and two (MEKK17 and RAF31_1) MAPKKK genes were induced and reached peak expression levels at 2 and 12 h after high temperature conditions (37 °C), respectively. Finally, nine MAPKKK genes were induced with peak expression at 4 or 6 h after the seedling leaves were cut with scissors, comprising ZIK1, five MEKKs (MEKK3_2, MEKK4_2, MEKK5_1, MEKK17 and MEKK20) and three Rafs (CTR1_1, RAF31_1 and RAF32).

In summary, 12 MAPKKK genes were induced by at least two of four signal-stress treatments. ZIK1, MEKK17, MEKK20 and RAF32 were induced and accumulated at higher levels after all signal-stress treatments. MEKK3_2, MEKK10_1, RAF30_1 and RAF38_3 were induced by three stress treatments. All 12 MAPKKK genes were induced by at least two of the five abiotic stress treatments. MEKK17 was induced by all five treatments, and ZIK1, MEKK3_2, MEKK10_1 and RAF32 were induced by four abiotic stress treatments. Two MEKKs (MEKK4_2 and MEKK20) and two Rafs (CTR1_1 and RAF31_1) were induced by three abiotic stress treatments, and four MAPKKK genes (MEKK5_1, MEKK17, RAF30_1 and RAF38_3) were induced by two abiotic stress treatments (Table 1).

**Table 1:** Expression profiles of the 12 investigated MAPKKK genes under different stress treatments in cotton.

Gene	Group	Signaling molecules				Environmental stress factors				
		JA (100 μM)	H_2_O_2_ (10 mM)	ABA (100 μM)	SA (10 mM)	Salt (200 mM)	PEG6000 (20%)	4 °C	37 °C	Wounding
ZIK1	ZIK	**	**	**	**	**	*	**	**	**
MEKK3_2	MEKK	**	**	*	**	**	*	**	**	**
MEKK4_2	MEKK	*	D	**	**	**	—	D	**	**
MEKK5_1	MEKK	**	D	**	D	**	*	*	D	**
MEKK10_1	MEKK	**	D	**	**	**	**	**	**	*
MEKK17	MEKK	**	**	**	**	**	**	**	**	**
MEKK20	MEKK	**	**	**	**	**	**	*	D	**
CTR1_1	Raf	**	**	*	*	D	**	D	**	**
RAF30_1	Raf	**	**	**	*	**	D	*	**	—
RAF31_1	Raf	*	D	**	**	**	*	—	**	**
RAF32	Raf	**	**	**	**	**	*	**	**	**
RAF38_3	Raf	**	*	**	**	**	*	*	**	—

Note: For various hormones and the environmental stress factors, the leaves of seedlings were sampled at 0, 0.5, 1, 2, 4, 6, 8, 10, 12 and 24 h after treatment. **and *indicate significant difference at P < 0.01 and P < 0.05, respectively. “—” represents undetected difference; “D” represents significant reduction for given MAPKKK gene expression after treatment. The Student’s *t*-test was performed between treated samples and mock controls.

### Identification of the complete MAPK signaling cascades

**Figure 9:**
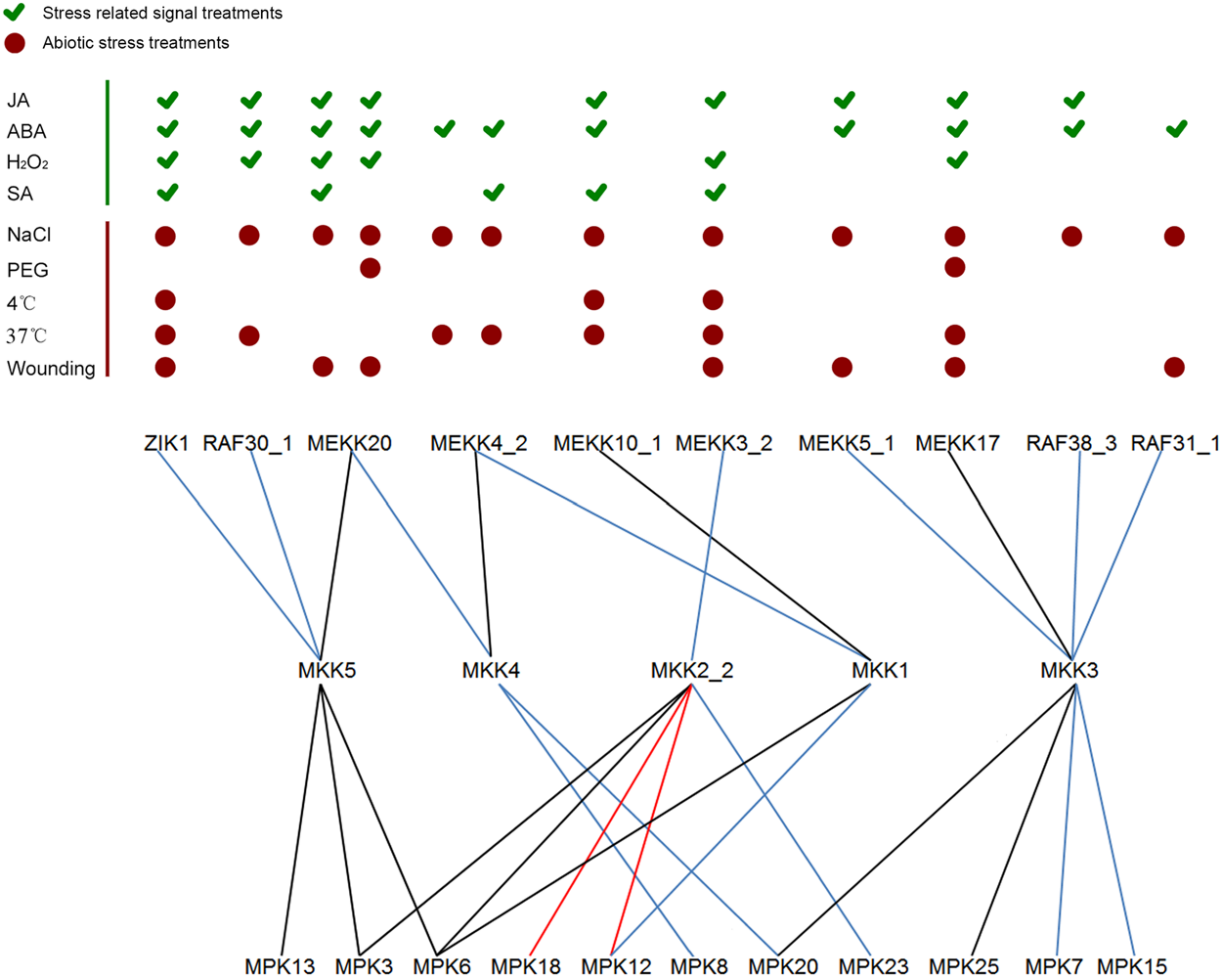
Integrated module of MAPK cascade in response to various treatments. The tick (green color) indicated the MAPK cascade commonly induced in stress-related signal treatments, including JA, ABA, H_2_O_2_ and SA. The circle (dark red) indicated the MAPK cascade commonly induced in stress treatment, including NaCl, PEG, 4 °C, 37 °C and wounding. Black lines indicated the interactions reported with confirmed function under stress in *Arabidopsis*, which was also identified in cotton. Red lines indicated the shared interactions in *Arabidopsis* and cotton, but no functions were reported in *Arabidopsis*. Blue lines indicated the interactions newly confirmed in cotton without reported in *Arabidopsis*.

To further characterize the potential function of the MAPK cascade, we constructed MAPK signaling networks by integrating data on the protein interactions and expression patterns of MAPKKK, MKK and MAPK genes. Due to low expression of MKK10_1 and the difficulty in detecting it in all tissues, we did not consider signaling cascades involving this protein. In total, we identified 38 potential MAPK signaling modules (Fig. 9). The members of each cascade showed similar responses to a given treatment. In the MEKK4_2/MEKK10_1-MKK1-MPK6/12 cascade, MEKK10_1, MPK6 and MPK12 were induced by JA, ABA, SA, NaCl, 4 °C and 37 °C, while MEKK4_2 was induced by JA, SA, NaCl and 37 °C. In the MEKK3_2-MKK2-MPK3/6/18/12/23 cascade, MEKK3_2 and five MAPK members were up-regulated by JA, H_2_O_2_, SA, NaCl, 4 °C, 37 °C and wounding. In the MEKK5_1/MEKK17/RAF38_3/RAF31_1-MKK3-MPK20/25/7/15 cascade, MEKK17 and four MAPKs were induced by JA, ABA, H_2_O_2_, NaCl, PEG, 37 °C and wounding; MEKK5_1 was induced by JA, ABA, NaCl and wounding; RAF38_3 was induced by JA, ABA and NaCl; and RAF31_1 was induced by ABA, NaCl and wounding. In the MEKK4_2-MKK4-MPK8/20 cascade, MEKK4_2, MPK8 and MPK20 were induced by ABA, NaCl and wounding. In the MEKK20-MKK4-MPK8/20 cascade, MEKK20, MPK8 and MPK20 were induced by JA, ABA, H_2_O_2_, SA, NaCl and wounding. In the ZIK1/MEKK20/RAF30_1-MKK5-MPK13/3/6 module, ZIK1, MPK13, MPK3 and MPK6 were up-regulated by JA, ABA, H_2_O_2_, SA, NaCl, 4 °C, 37 °C and wounding; MEKK20 was induced by JA, ABA, H_2_O_2_, SA, NaCl and wounding; and RAF30_1 was induced by JA, ABA, H_2_O_2_, NaCl and 37 °C. These complete MAPK signaling cascades indicate the important role of post-transcriptional regulation of MAPK cascades and the complicated cross-talk between signal and abiotic stresses, laying a foundation for understanding the functional roles of different MAPK cascades in signal transduction pathways, and for the improvement of various defense responses in cotton.

## Discussion

Genome-wide analysis of distinct modules in MAPK cascades has been characterized in several plant species. A total of 20 MAPKs, 10 MAPKKs, and 80 MAPKKKs have been reported in *Arabidopsis*^2,6^; and 17 MAPKs, 8 MAPKKs and 75 MAPKKKs in *O*. *sativa*^7–9^. In cotton, 28 MAPKs^33^ and 11 MKKs^32^ have been analyzed, and 89 MAPKKKs were identified in *G*. *raimondii* in the study. Unlike the high amino acid sequence identity of orthologous MKKs and MAPKs in other plant species, MAPKKKs in cotton showed high sequence diversity, especially outside the kinase domain. These regions comprise long C or N-terminal regions that as a scaffold to activated MAPKKs or other proteins in signaling cascades. Consistent with *Arabidopsis* and rice, MAPKKK family members in *G*. *raimondii* were classified into three groups according to their phylogenetic clades, the MEKK, Raf and ZIK subfamilies, indicating that MAPKKKs expanded before the split of monocots and eudicots. The conserved exon numbers in each subgroup supported their close evolutionary relationship, however, the divergent gene structures among different phylogenetic subgroups suggested that MAPKKK genes might have developed in ancient times and that offspring genes evolved diverse exon/intron structures, possibility to accomplish different functions in the *G*. *raimondii* genome. In addition, synteny comparison indicated that the MAPKKK family members in the *G*. *raimondii* genome mainly resulted from segmental duplication events, which is consistent with the development of the MKK and MAPK families^32,33^.

Several MKK and MAPK genes have been characterized in many species, whereas only a limited number of MAPKKK genes have been functionally characterized in plant growth and development. In *Arabidopsis*, ANP2 and ANP3 are involved in the regulation of cytokinesis^20^. YDA has been shown to negatively regulate stomatal development^21^. MAPKKKδ4 participates in regulating shoot branching and growth^41^. Raf5 functions in controlling sugar resistant seedling development^42^. *OsMAPKKK43* plays the important role in mechanical tissue formation at the leaf lamina joint^26^. In our study, the expression of MAPKKKs was investigated in different *G*. *hirsutum* tissues/organs. Most of the MAPKKKs in the same subgroup shared similar expression patterns, for example, MEKK13_1, MEKK13_2, MEKK14_1 and MEKK14_2 had higher expression levels in petals and anthers than other organs. However, some MAPKKKs in the same subgroup had different expression patterns, indicating their functional divergence during plant growth and development. In addition, some MAPKKKs in different groups also showed similar expression patterns, such as MEKK21_1, RAF39_4 and ZIK6_1 with higher expressions in 20 days post-anthesis (dpa) fibers than in other organs, suggesting that the different groups of MAPKKKs may have similar functions or are involved in the same signaling pathways.

In *Arabidopsis*, MEKK1 plays an important role in oxidative, salt and cold stress signaling, and defense responses^19,43,44^. MEKK17 and MEKK18 have been reported to act on MKK3 to mediate ABA signaling^31^. CTR1 is an essential component of ethylene (ET) signaling, and EDR1 is an essential component of SA and ET stress signaling and is involved in plant innate immunity^22,23^. Raf5 plays a negatively regulated role in salt tolerance^24^. In rice, the ACDR1 (accelerated cell death and resistance 1) and DSM1 mutant1 (drought-hypersensitive) are Raf-like MAPKKKs, and play an important role in response to fungal pathogen and drought stress, respectively^25,45^. Although functional analysis of ZIK-like genes is limited, most rice ZIK-like genes have been shown to be up-regulated by at least one abiotic stress, suggesting that they might be response to abiotic stress^7^. In tomato, *SlMAPKKKα* and *SlMAPKKKε* are also reported to participate in plant innate immunity^27,46,47^. Research into the MAPKKK gene family in tomato^48^, rice^7^, maize^10^, *vitis vinifera*^39^, canola^5^, wheat^49^, cucumber^11^ and *Brachypodium distachyon*^12^ have shown that its members are involved in signal and abiotic stress responses. In accordance with previous reports in other species, the 12 MAPKKK genes investigated in the present study in cotton are generally responsive to stress-related signal and abiotic stress treatments. In detail, all of the MAPKKK genes investigated were induced by at least two stress-related signal treatments, and 33.3% were induced by four or three stress-related signal inducers, respectively. All of the MAPKKK genes investigated were induced by at least two abiotic stresses. MEKK17 was induced by five abiotic stresses, and 33.3% and 41.6% were induced by four and three abiotic stresses, respectively. The present results show that MAPKKK genes play a crucial role in responses to environmental stresses in cotton, and highlight the cross-talk between stress-related hormones and abiotic stresses.

MAPKs modules can interact with upstream and downstream protein components to regulate numerous physiological responses. As the first member of MAPK cascades, MAPKKKs may participate in multiple signal transduction pathways and regulate various biological processes^1,5,50^. Up to now, no complete MAPK cascades have been reported in cotton. In this study, based on Y2H assays with three biological replicates, a total of 18 interaction pairs of MAPKKKs/MKKs were identified in cotton. Of them, 13 were novel compared with that in *Arabidopsis*, implying the inherent difference between *Arabidopsis* and cotton in signaling pathways. When data on MKK-MAPK and MKK-MAPKKK protein interactions were combined with data on their expression patterns, 38 potential MAPK signaling modules were identified in cotton.

Previous studies indicated that hormones played crucial roles in adapt to changing environments, and the coordinated regulation of hormone biosynthetic pathways played key roles in the adaptation of abiotic stress^51,52^. In this study, we detected the members with protein interaction in MAPK cascades showed the similar expression induced by stress-related hormones and abiotic stresses. Further, SA can regulate response to biotrophic pathogens and systemic acquired resistance, while JA mediates response to necrotrophs, JA also has an important role in SAR and response to wounding^53,54^. H_2_O_2_ can induce oxidative bursts, which may contribute to resistance abiotic stress^55^. ABA mediates the signaling pathway in response to cold, salt and drought stresses^56^. We detected that the MEKK4_2/MEKK10_1-MKK1-MPK6/12 cascade was induced by ABA, SA, NaCl and 37 °C. The MEKK10_1-MKK1-MPK6 cascade in cotton corresponded to the MEKK10-MKK1-MPK6 cascade in *Arabidopsis*, which has previously been reported to function in the response to salt and cold stress and pathogen attack in *Arabidopsis*^19,44,57^. In addition, GhMKK1 plays important roles in response to salt, drought and pathogen attack^58^, GhMPK6 is involved in ABA signaling pathways and induced CAT1 expression and H_2_O_2_ production^59^, suggesting that the cascade might be responsible in salt and drought stress and defense against pathogens. In the MEKK3_2-MKK2-MPK3/6/18/12/23 cascade, MEKK3_2 and five MAPKs were induced by JA, H_2_O_2_, SA, NaCl, 4 °C, 37 °C and wounding. GhMKK2 was reported to be involved in pathogen attack^60^, and GhMPK11 (a paralog of GhMPK18) has been shown to be negatively regulated in response to pathogen attack^61^. In the MEKK5_1/MEKK17/RAF38_3/RAF31_1-MKK3-MPK20/25/7/15 cascade, four MAPKKK and four MAPK members were induced by ABA and NaCl, especially MEKK17, and four MAPK members were induced by JA, ABA, H_2_O_2_, NaCl, PEG, 37 °C and wounding. Further, GhMKK3 played an important role in tolerance to drought by regulating stomatal development and root growth^62^. The MEKK17/18-MKK3-MPK1/2/7 cascades in *Arabidopsis*, which was reported to be involved in the ABA signaling pathway, and mediated plant growth by regulating the timing of senescence in an ABA-dependent manner^31^. GhMPK7 (a paralog of GhMPK20 and GhMPK25) was involved in disease resistance and plant development, and *GhMPK25* has been characterized in response to *V*. *dahlia*^33^, suggesting that this MAPK cascade may be involved in responses to drought and pathogens in cotton. In addition, MEKK17, MEKK19, RAF31_1 and RAF38_3 were also found to interact with MKK3; however whether these MAPKKKs are involved in the ABA signaling pathway through the MKK3-MPK5/8/14/20/23/25 module needs to be further investigated. In the MEKK4_2-MKK4-MPK8/20 cascade, GhMKK4 was reported previously to play an important role in ABA, gibberellin (GA) and H_2_O_2_ signaling^63^, while YDA (MEKK4) in *Arabidopsis* participates in regulating stomatal development. MEKK4_2, MPK8 and MPK20 were also induced by ABA, SA, NaCl and wounding, which suggests that the cascade may be involved in NaCl and stomatal responses. In the ZIK1/MEKK20/RAF30_1-MKK5-MPK13/3/6 cascade, MEKK20, MPK13, MPK3 and MPK6 were up-regulated by JA, ABA, H_2_O_2_, SA, NaCl and wounding. GhMKK5 was induced by abiotic stresses, pathogen infection and multiple defense related signal molecules^64^. The AIK1 (MEKK20)-MKK5-MPK6 cascade was triggered in response to ABA, and regulated the stomatal responses and root growth in *Arabidopsis*^65^. Taken together, the results of this systematic investigation of the complete MAPK signaling cascades indicate that conserved MAPK cascades serve as a bridge for hormones and abiotic stresses, which will enhance our understanding of MAPK signal transduction pathways and utilize them in improving cotton development and stress-tolerance breeding.

## Conclusions

We identified 89 MAPKKK genes in *G*. *raimondii*, and found that segmental duplication played a central role in the expansion of the MAPKKK family in the *G*. *raimondii* genome. GrMAPKKKs could be phylogenetically classified into three subgroups. MAPKKKs showed a variety of expression patterns in vegetative and reproductive organs, and were involved in a diverse range of signaling pathways following treatment with hormones or abiotic stresses. When data on MKK/MAPK and MKK/MAPKKK interaction pairs were combined with their expression patterns, 38 potential MAPK signaling modules were identified as being involved in the complicated cross-talk between signal and abiotic stress pathway components. Our work first systematically elucidated the relationships of MAPK cascade proteins and will lay a foundation for understanding their functions and utilizing these signal pathways in cotton breeding.

## Materials and Methods

### Characterization and analysis of the MAPKKK genes in *G*. *raimondii*

The genomic databases of *G*. *raimondii*, *A*. *thaliana* and *O*. *sativa* were downloaded from Phytozome v9.0 (http://www.phytozome.net/). The gene database of three cotton species, *G*. *arboreum*, *G*. *hirsutum* acc. TM-1, and *G*. *barbadense* acc. 3–79, were downloaded from http://cgp.genomics.org.cn, http://mascotton.njau.edu.cn and http://cotton.cropdb.org, respectively. The Pfam protein family database (http://pfam.sanger.ac.uk) with the MAPKKK domain (PF00069) and HMMER software version 3.0^66^ were used to search for putative MAPKKK proteins against the four cotton species. The sequences of the conserved domains were further confirmed using SMART^67^ and INTERPROSCAN^68^.

Gene nomenclature of MAPKKKs in *G*. *raimondii* was following its homology with *Arabidopsis* MAPKKK proteins. If two or more genes have the same homolog in *Arabidopsis*, they were distinguished by an extra number. Further, the corresponding orthologs in *G*. *arboreum*, *G*. *hirsutum* acc. TM-1, and *G*. *barbadense* acc. 3–79 were named using the same number for orthologs as in *G*. *raimondii* (Supplementary Dataset 1).

The ExPASy proteomics server (http://expasy.org/) was used to predict MAPKKK isoelectric points and molecular weights. The CELLO v2.5 server (http://cello.life.nctu.edu.tw/) was used to analyze the subcellular localization of MAPKKKs. MapInspect (http://www.plantbreeding.wur.nl/UK/software_mapinspect.html) was used to map MAPKKK genes, and the Plant Genome Duplication Database (PGDD; http://chibba.agtec.uga.edu/duplication/) was used to identify syntenic information in *G*. *raimondii*. Phylogenetic tree was constructed using MEGA6 software with the Maximum likelihood method. The online program Gene Structure Display Server (GSDS; http://gsds.cbi.pku.edu.cn/) was used to draw the exon and intron structures. Paralogous pairs of nonsynonymous to synonymous substitutions (Ka/Ks) were analyzed to assess their selection pressure in the evolutionary process. Generally, Ka/Ks <1 indicates negative or purifying selection; Ka/Ks = 1 indicates neutral selection; and Ka/Ks >1 indicates positive selection^69^.

### Expression of MAPKKK genes in *G*. *hirsutum*

The RNA sequencing data of *G*. *hirsutum* acc TM-1 were download from http://mascotton.njau.edu.cn and the NCBI database (SRA: PRJNA248163). Fragments per kilobase of exon model per million mapped reads (FPKM) was used to calculate the expression levels of MAPKKK genes with the Z-score formula: z = (x-μ)/σ. In this equation: x = FPKM values of MAPKKK in one tissue; μ = average values of MAPKKK in eight tissues; σ = standard error of MAPKKK values in eight tissues.

### Yeast two-hybrid assays

The coding sequences of MAPKKKs were amplified from *G*. *hirsutum* acc. TM-1 using gene-specific primers with *Eco*RI/*Bam*HI enzyme sites, and were cloned into pGADT7 vectors (Supplementary Dataset 5). Combined with eight MKKs-PGBDT7 vectors constructed, and the auto-activation of eight MKK proteins was tested previously^32^, 51 MAPKKKs and eight MKK proteins were co-transformed into yeast strains Y2H Gold according to the manufacturer’s instructions (Clontech, Mountain View, CA, USA). Colonies were tested on selective medium [SD/-Trp/-Leu and SD/-Trp/-Leu/Aureobasidin A (AbA)], and transferred to SD/-Ade/-His/-Leu/-Trp medium and supplemented with X-α-Gal and AbA for 7days at 30 °C, only yeast colonies with interactions between MKKs and MAPKKKs were able to grow on the selection media. The vectors, pGBKT7–53 and pGADT7-rec, were used as positive control, and pGBKT7-Lam and pGADT7-rec as negative control. All interactions were further verified and confirmed with three biological replicates.

### Plant materials and treatments

*G*. *hirsutum* cv. Jinmain 19 was used for different treatments, including signaling stress (100 μM JA, 100 μM ABA, 10 mM SA or 10 mM H_2_O_2_), abiotic stress (200 mM NaCl, 20% PEG, 37 °C high temperature, 4 °C low temperature and wound treatment). The treatments were following the methods described previously^32^. All tissues were collected and stored at −70 °C after being flash-frozen in liquid nitrogen. All necessary permits for collecting *G*. *hirsutum* cv. Jinmain 19 were obtained from Nanjing Agricultural University, Jiangsu Province, China.

### Extraction of total RNA

Total RNA was extracted from cotton leaves using a Biospin plant Total RNA extraction kit (BioFlux) according to the manufacturer’s protocol. First strand cDNA was synthesized using the Superscript first-strand synthesis system (Invitrogen, Foster City, CA). The primers for cotton MAPKKKs were designed by Beacon Designer 7.0 (Reference gene: histone3). Real-time PCR was done using SYBR Green (Roche, Switzerland) and performed on an ABI7500 Real time PCR system (Applied Biosystems, USA). The PCR program was as follows: initial denaturation at 95 °C for 10 min, 40 cycles of denaturation at 95 °C for 15s, 60 °C for 15s and 72 °C for 15s. Expression levels of MAPKKK genes were calculated according to methods described by Livak and Schmittgen^70^.

### Statistical analysis

All experiments were repeated independently at least three times. Data obtained were subjected to statistical analysis using student’s *t*-tests and probability values of P < 0.05 were considered as significant between the different treatments.

### Availability of data and materials

MAPKKK genes were identified from the four sequenced cotton species, including *G*. *raimondii*, *G*. *arboreum*, *G*. *hirsutum* acc. TM-1, and *G*. *barbadense* acc. 3–79. The genomic database could be obtained from http://www.phytozome.net/, http://cgp.genomics.org.cn, http://mascotton.njau.edu.cn/, and http://cotton.cropdb.org/cotton/, respectively. All the cloned sequences were uploaded into NCBI GenBank under the accession numbers: MF150710-MF150760.

## Electronic supplementary material

Supplementary information

Dataset 1

Dataset 2

Dataset 3

Dataset 4

Dataset 5
