# Supplementary material for: Identification on mitogen-activated protein kinase signaling cascades by integrating protein interaction with transcriptional profiling analysis in cotton
Source: Sci Rep. 2018 May 25;8:8178. doi: 10.1038/s41598-018-26400-w (PMC5970168; doi:10.1038/s41598-018-26400-w)
Supplement: Supplementary file 1 — Supplementary information [file 41598_2018_26400_MOESM1_ESM.pdf]

# Identification on mitogen-activated protein kinase signaling cascades by integrating protein interaction with transcriptional profiling analysis in cotton

**Xueying Zhang, Xinyue Mi, Chuan Chen, Haitang Wang, Wangzhen Guo***

**State Key Laboratory of Crop Genetics & Germplasm Enhancement, Hybrid Cotton R & D Engineering Research Center, Ministry of Education, Nanjing Agricultural University, Nanjing 210095, China**

*** Correspondence and requests for materials should be addressed to W.Z.G (email: moelab@njau.edu.cn)**

**Supporting Information**

**Supplementary Figure 1.** The conserved motif and domains of MAPKKK genes in *G. raimondii*.

**Supplementary Figure 2.** Phylogenetic analysis, intron-exon identification and structural comparison of 89 MAPKKK genes in *G. raimondii.*

**Supplementary Figure 3.** Expression patterns of the 12 MAPKKK genes in response to stress-related signal treatments (JA, $H_2O_2$, ABA and SA).

**Supplementary Figure 4.** Expression patterns of the 12 MAPKKK genes in response to stress treatments (NaCl, PEG, 4℃, 37℃ and wounding).

**Supplementary Figures**

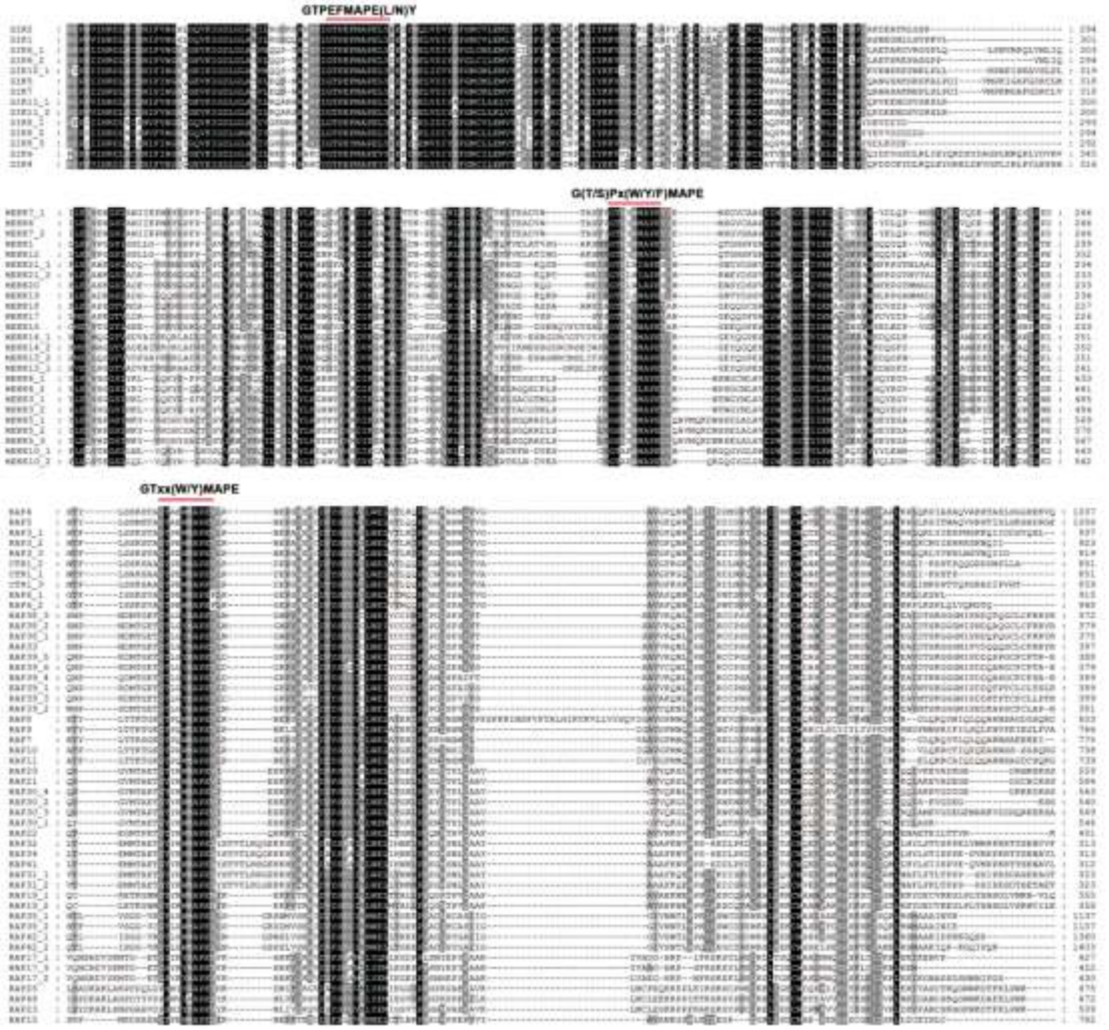


**Supplementary Figure 1. The conserved motif and domains of MAPKKK genes in *G. raimondii*.**

The conserved GTPEFMAPE(L/V)Y motif in the ZIK subfamily, G(T/S)PX(W/Y/F)MAPEV motif in the MEKK subfamily, and GTXX(W/Y)MAPE motif in the Raf subfamily, were highlighted with red underline.

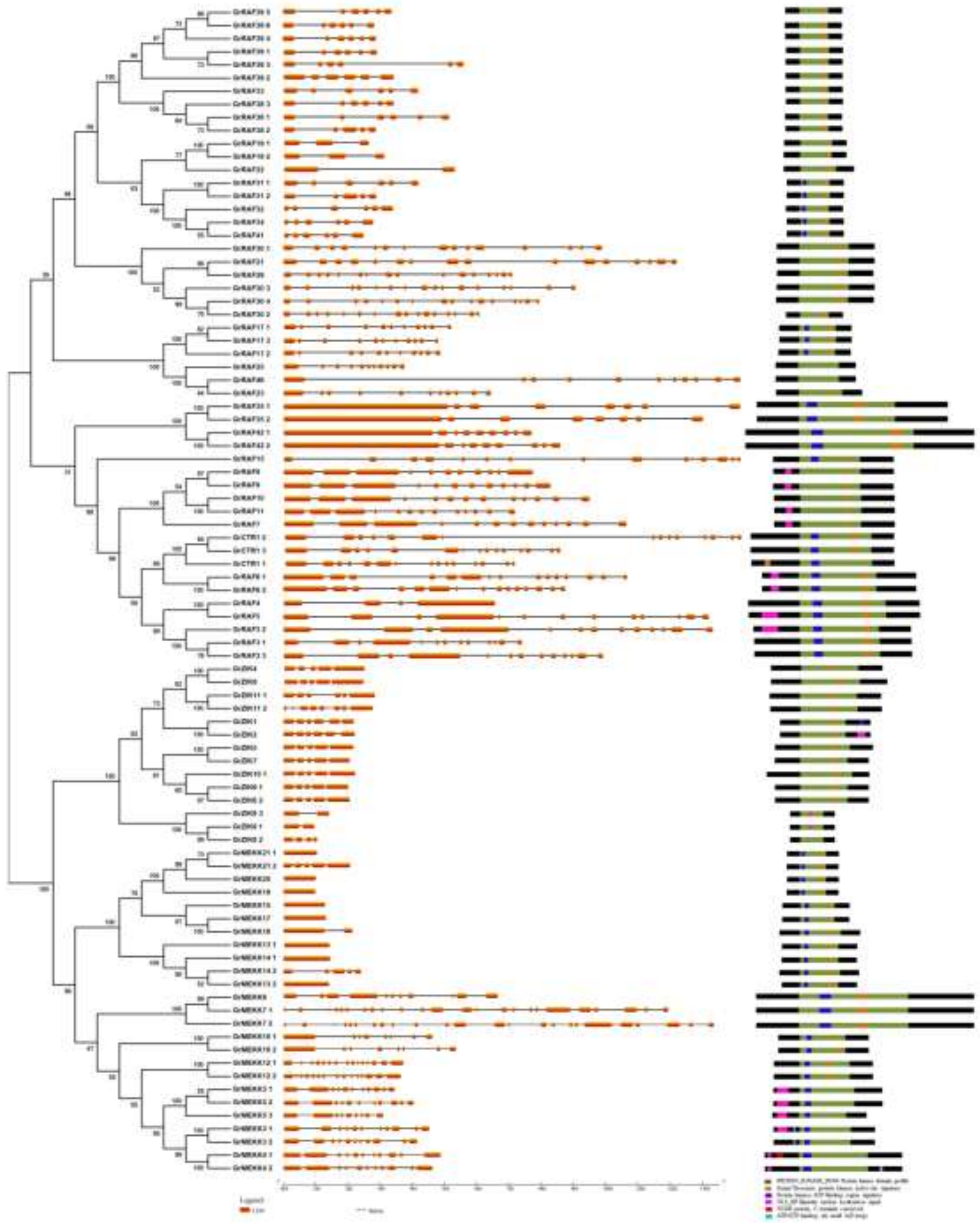

**Supplementary Figure 2. Phylogenetic analysis, intron-exon identification and structural comparison of 89 MAPKKK genes in *G. raimondii*.**

Phylogenetic tree was constructed using Maximum likelihood (ML) method with 1000 bootstrap replicates. Introns and exons were represented by black line and orange box, respectively. Four conserved domains of each MAPKKK were shown with light blue, orange, green and dark blue, respectively.

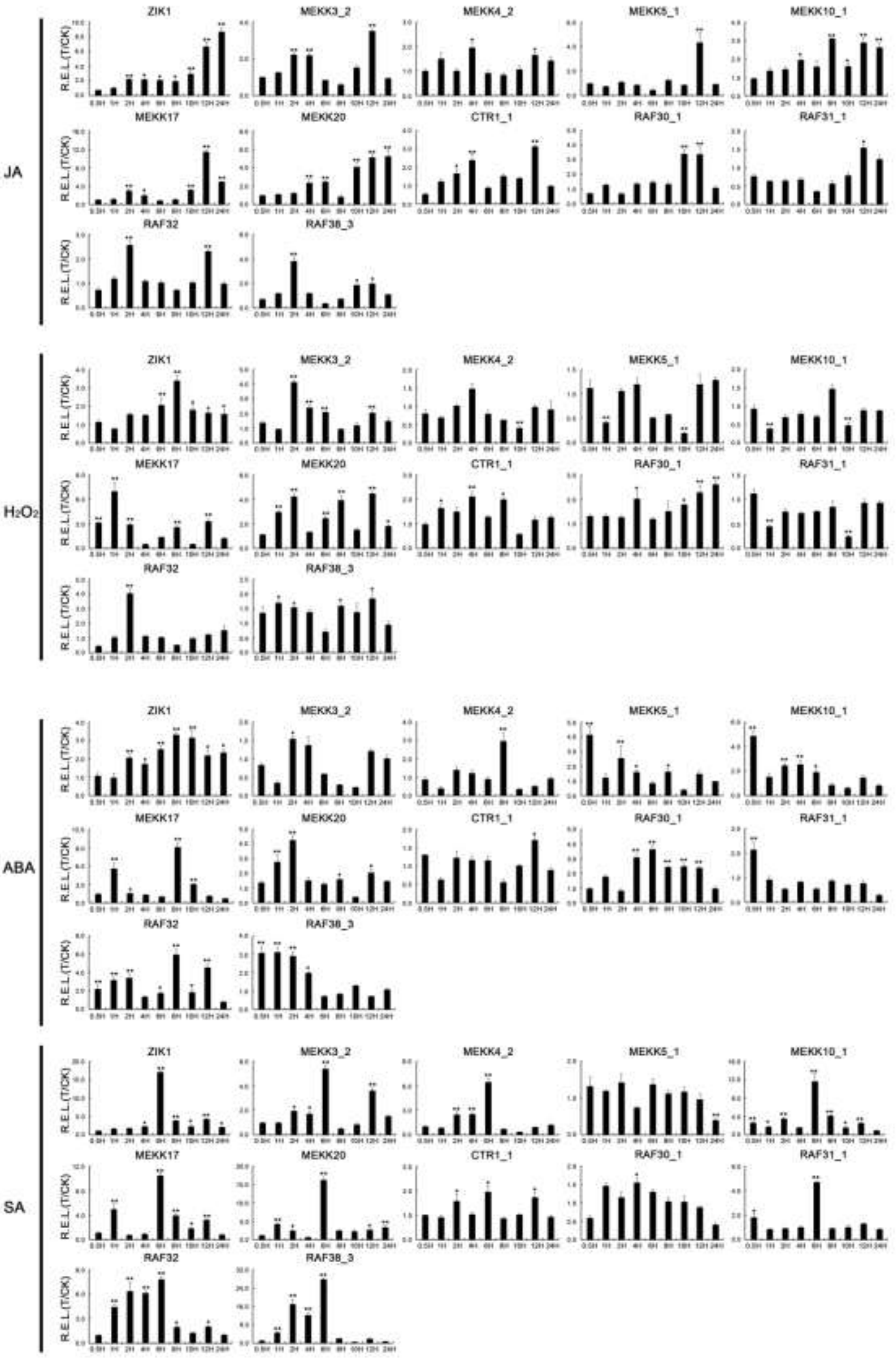


**Supplementary Figure 3. Expression patterns of the 12 MAPKKK genes in**

**response to stress-related signal treatments (JA, $H_2O_2$, ABA and SA).**

The expression patterns were presented as the mean fold by comparing treated samples with controls. X-axis: hours of signal treatments; Y-axis: relative expression levels. The error bars indicated standard deviation of three biological replicates (“*” $p<0.05$; “**” $p<0.01$).

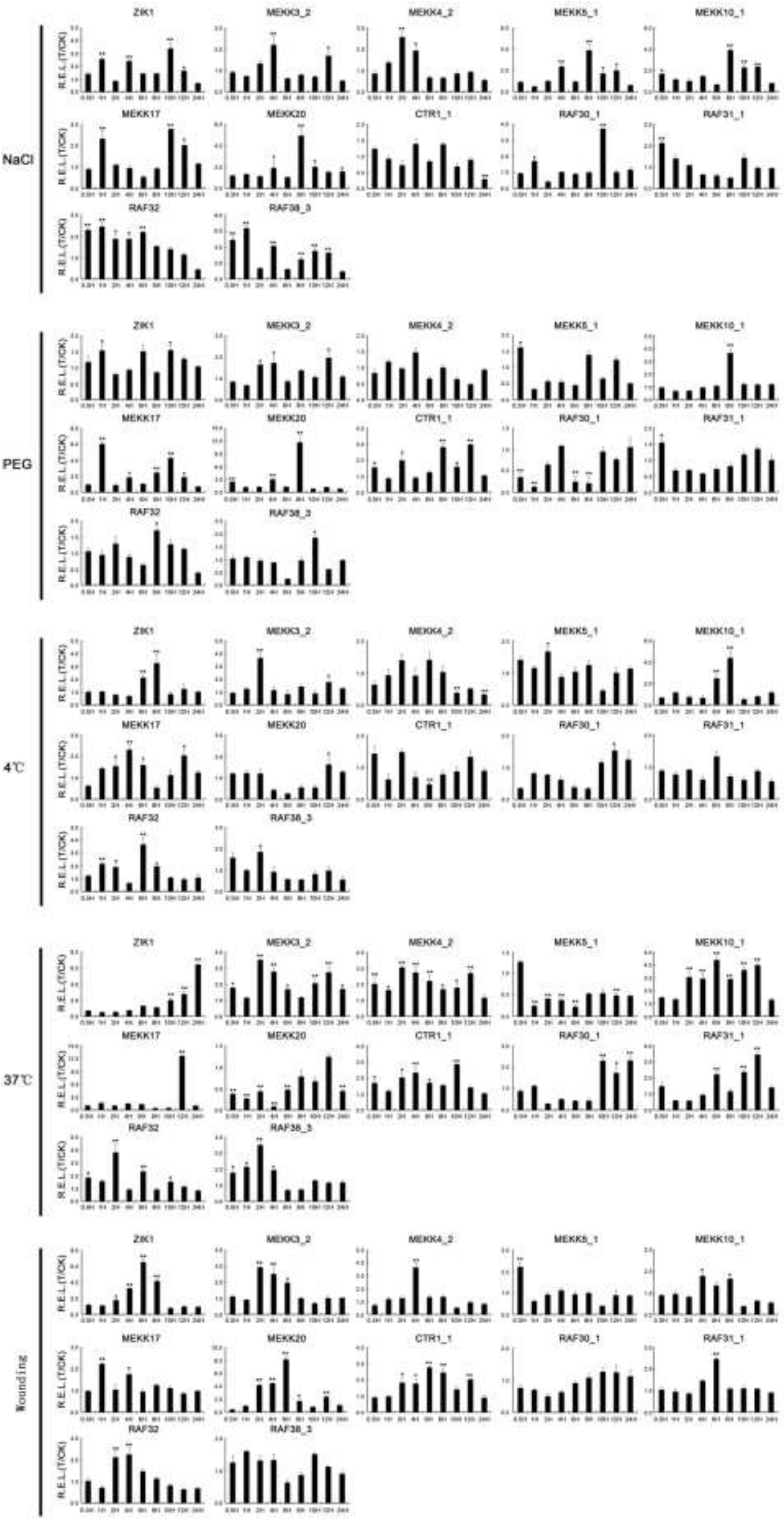
NaCl
PEG
4℃
37℃
Wounding
ZIK1
MEKK3_2
MEKK4_2
MEKK5_1
MEKK10_1
MEKK17
MEKK20
CTR1_1
RAF30_1
RAF31_1
RAF32
RAF38_3
R.E.L (T/CK)

**Supplementary Figure 4. Expression patterns of the 12 MAPKKK genes in response to stress treatments (NaCl, PEG, 4℃, 37℃ and wounding).**

The expression patterns were presented as the mean fold by comparing treated samples with controls. X-axis: hours of stress treatments; Y-axis: relative expression levels. The error bars indicated standard deviation of three biological replicates (“*” $p<0.05$; “**” $p<0.01$)
